# Exploring Gambling-like Behaviors in Children’s Cigarette Card Games and Parental Pro-game Perceptions in China: A Qualitative Study

**DOI:** 10.1007/s10899-026-10514-2

**Published:** 2026-05-29

**Authors:** Xue Weng, Yongfang Deng, Yufei Xia, Jiashuo Wang, Sophia Siu-Chee Chan, Sheng Zhi Zhao

**Affiliations:** 1 Research Center for Health and Social Policy, Beijing Normal University, Zhuhai, China; 2 Obstetric department, Guangzhou Women and Children’s Medical Center, Guangzhou Medical University, Guangzhou, China; 3 School of Sociology, Beijing Normal University, Beijing, China; 4 School of Public Health, The University of Hong Kong, Hong Kong, Hong Kong

**Keywords:** Gambling-related behavior, Cigarette card game, Parental facilitation, Tobacco normalization, Qualitative study

## Abstract

Cigarette card games emerged as a popular yet controversial pastime among children in China, raising concerns on the potential to foster gambling-like behaviors. This study explored children’s experiences and perceptions of cigarette card games, as well as parental attitudes and facilitation practices. Semi-structured interviews were conducted with 21 child-parent dyads (19 boys, 2 girls) from September to December 2024. Purposive and snowball sampling techniques were adopted to recruit children who had played cigarette card games in the past 1 month. Separate interview protocols were developed for children and parents to ensure age-appropriate communication. Interviews were audio-recorded, transcribed verbatim, and analyzed thematically following Braun and Clarke’s framework, with dual coding and triangulation. Four key themes emerged: (1) Chance and reward-driven engagement, where children linked the thrill of obtaining rare cards to gambling; (2) Peer influence and social reinforcement, which strengthened friendships but risked excluding non-players; (3) Parental misperceptions, viewing the games as healthier alternatives to screen time that promote outdoor activity; and (4) Parental facilitation, including sourcing cards through networks and rationalizing support as aiding socialization. These patterns indicate that cigarette card games may expose children to gambling-like reward systems and normalize tobacco-related imagery, with parental participation further amplifying such exposure and acceptance. Cigarette card games blend gambling-like features with early tobacco normalization, amplified by parental pro-game practices. Public health interventions should target both children and parents, promoting awareness of gambling risks and guiding families toward safe, prosocial forms of play.

**Supplementary Information:**

The online version contains supplementary material available at https://doi.org/10.1007/s10899-026-10514-2.

## Key Practitioner Message

• Cigarette card games are a popular form of play among children in China, incorporating chance and reward elements that resemble gambling. Parents often view these games as harmless and socially beneficial, overlooking potential risks.

• Our findings show that children display gambling-like excitement and risk-taking, while parental encouragement reinforces reward-seeking behaviors and normalizes exposure to tobacco imagery.

• These results reveal an overlooked pathway for early gambling-like socialization and tobacco normalization. Practitioners should educate parents about these hidden risks and promote safer, prosocial play alternatives.

**Supplementary Information:**

The online version contains supplementary material available at https://doi.org/10.1007/s10899-026-10514-2.

## Introduction

**Fig. 1:**
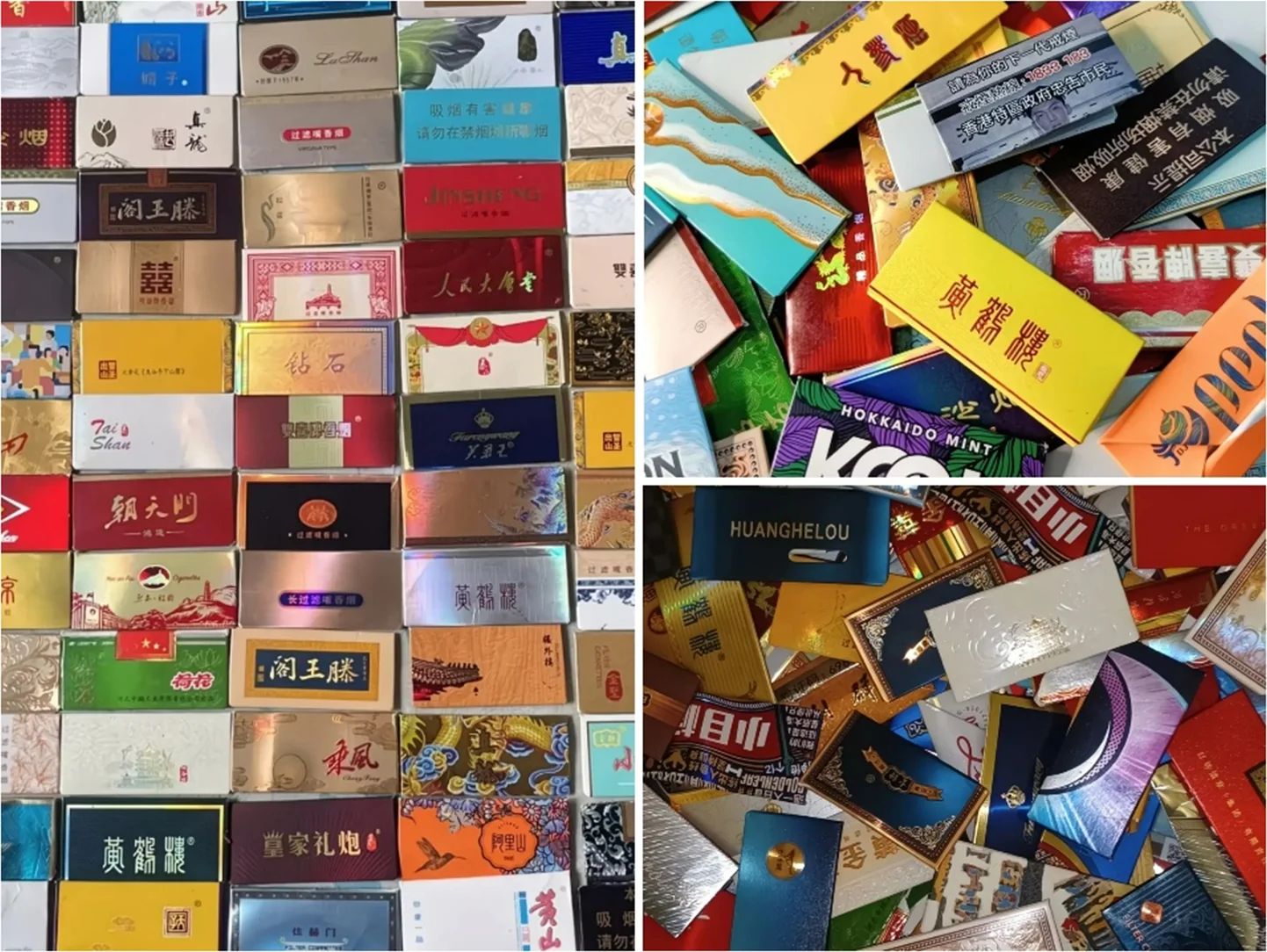
Sample cigarette cards, cut and folded from discarded cigarette packs

Despite notable progress in tobacco control since China’s ratification of the WHO Framework Convention on Tobacco Control (FCTC) in 2005, China remains the world’s largest producer and consumer of tobacco (Chan et al., 2023). Smoking continues to permeate daily life, functioning not only as a personal habit but also as a cultural symbol of social connection and reciprocity (Chan et al., 2023). Smoking remains highly prevalent among Chinese adults, particularly men, with national data indicating that approximately half of men smoke (Chan et al., 2023). Smoking among children and adolescents is nevertheless socially disapproved and prohibited in China. Tobacco sales to minors are banned, and schools commonly enforce anti-smoking policies. Yet tobacco branding remains visible in everyday life, including on cigarette packaging, creating continued exposure to tobacco-related imagery despite these formal restrictions. Within this enduring context of tobacco normalization, cigarette cards made from discarded packs have recently gained rapid popularity among primary school-aged children (aged 6–12 years) (Jiang, 2024). As shown in Fig. 1, these cards are typically folded into triangular or rectangular shapes featuring tobacco brand logos, design elements, and packaging aesthetics. The visual appeal of rare cards from premium brands (e.g., Chunghwa’s distinctive red and gold branding) drives demand and creates a social hierarchy among players (Zhao et al., 2025). In common formats, players wager their cards by throwing them to land face-up or flip opponents’ cards. Some play may be relatively light-hearted, but when children keep the cards they win, the game becomes a wager-based competition in which they risk their own cards for the chance to gain others’ cards (Jiang, 2024; Lin et al., 2025). The phenomenon has been amplified by social media and short-video platforms such as TikTok, where tutorials and gameplay videos attract large audiences and have accelerated the nationwide spread of the game since 2024 (Chen, 2024; Tone, 2024).

Previous studies have highlighted the potential risks of tobacco exposure and early smoking experimentation among children participating in cigarette card games (Jiang, 2024; Zhao et al., 2025). These games incorporate elements of chance and randomness that mirror gambling mechanisms such as slot machines and loot boxes, which are digital game features involving randomized rewards whose contents are unknown until opened (Drummond et al., 2020; Ferrari et al., 2018; Spicer et al., 2022). Although cigarette card games differ in that children usually know what cards they have obtained, they similarly involve uncertainty during gameplay, as children risk their own cards for the chance of winning more, with outcomes largely dependent on luck rather than skill (Yau & Potenza, 2015). The appeal often centers on the prospect of acquiring rare, high-valued cards from premium tobacco brands like *Chunghwa*, which can create addictive reward cycles through the thrill of uncertain wins, social recognition tied to card collections, and the ongoing desire to expand collections. Because the cards are often sourced through scavenging, trading, or online purchasing, the gameplay can encourage risk-taking and loss-chasing behavior resembling those seen in gambling trajectories (Xiao et al., 2024). Prior research has linked such chance-based reward systems to problem gambling and associated mental health issues (Xiao et al., 2024), both of which are increasingly prevalent among young people (Reynolds et al., 2023; Tran et al., 2024).

Global efforts under the WHO FCTC call for protecting minors from exposure to tobacco marketing and branding, as early exposure significantly increases the likelihood of tobacco initiation (Montini et al., 2010). Collectible items like cigarette cards represent a subtle yet influential form of tobacco promotion, echoing historical tobacco industry strategies that used branded merchandise to cultivate brand familiarity and loyalty among youth (Peters et al., 2015; Pollay, 2000). By combining elements of chance, rewards, and competition, cigarette card games may foster addiction-like behaviors in children while reinforcing the appeal of tobacco imagery. Socialization processes, particularly social media exposure and parental perceptions, are positively shaping children’s gambling attitudes, behavior, and future gambling intentions (Lapierre et al., 2017; Pitt et al., 2017). Many parents underestimate the potential harm, often providing children with cigarette packs or assisting in acquiring cards from others or online sources (Chen, 2024). Parental perceptions are therefore central to sustaining children’s engagement (Botella-Guijarro et al., 2020; Canale et al., 2016; Pitt et al., 2017). A recent population-based survey in Shenzhen found that 21.5% of parents viewed these games as low risk for smoking initiation, and 44.3% expressed tolerance toward children’s gameplay (Lin et al., 2025). Given that parental facilitation is a well-documented predictor of adolescent smoking initiation (Gilman et al., 2009) and problem gambling (Zhai et al., 2017), such tolerance may unintentionally normalize both tobacco use and gambling-related behaviors among children.

Despite advancements in understanding tobacco marketing and parental perceptions (Chen-Sankey et al., 2022), qualitative evidence on children’s experiences with cigarette card games and the influence of parental perceptions on their participation remain limited. Our previous qualitative research has examined the appeal of cigarette packaging and advocated for stricter regulation (Zhao et al., 2025). However, how these games cultivate gambling-like behaviors and how parental pro-game perceptions influence children’s participation remain underexplored. Understanding this issue is crucial to explain the social and psychological processes that sustain children’s engagement and to inform policy efforts that mitigate the harms associated with early exposure to gambling-like behaviors. This study addressed two research questions. First, in what ways do children’s experiences of cigarette card games reflect gambling-like behaviors? Second, how do parents perceive these games, and how do such perceptions shape their tolerance of, or facilitation of, children’s participation?

## Methods

### Study Design

We conducted semi-structured, in-depth interviews with primary school children and their parents, treated as matched dyads, to explore the family engagement in cigarette card games. This dual-perspective design allowed for cross-validation by integrating children’s experiences with parental perceptions and facilitation practices. To encourage candid responses and minimize social desirability bias, children and parents were interviewed separately. Data collection took place from September to December 2024, a period marked by intense media coverage and rising popularity of cigarette card games in China since April 2024, providing a timely context for exploring emergent behaviors. Ethics approval was obtained from the Institutional Review Board of Beijing Normal University (SSDPP-HSC-2024005).

### Sampling and Recruitment

Eligible participants included children aged 6–14 years (primary school age) who had engaged in cigarette card games within the last month, one parent who was familiar with the child’s gameplay and daily routines. This age range was selected because cigarette card games have been reported primarily among primary school-aged children in China, whereas adolescents are less commonly involved. We aimed to capture a heterogeneous sample across socioeconomic and regional backgrounds in China. A total of 21 child-parent pairs (19 boys and 2 girls; 17 mothers and 4 fathers) were recruited through purposive sampling methods complemented by snowball sampling. Recruitment channels included school networks, extracurricular activity groups, and social media platforms such as WeChat. Parents completed a brief questionnaire on their child’s gameplay and household characteristics to ensure diversity across family contexts. Recruitment continued until no new themes emerged, with thematic saturation achieved after 19 dyads and two additional pairs interviewed to confirm completeness.

### Data Collection

A total of 42 interviews (21 children and 21 parents) were conducted separately, guided by open-ended interview protocols designed specifically for each group. The interview domains were informed by the study aims and existing literature on cigarette card games, gambling-like behaviors, and parental perceptions. For children, visual prompts (e.g., images of cigarette cards and gameplay scenarios) and simplified language were used to facilitate understanding. Topics included motivations for playing, strategies for playing and gambling elements, sources of cards, peer engagement, and family attitudes. Parent interviews explored attitudes towards the children’s gameplay, perceived benefits and harms, household smoking behaviors, and pro-game practices that might facilitate gameplay, such as providing cigarette packs or assisting in online card purchases. Separate interviews allowed children to express themselves freely without parental influence. Cross-validation was achieved by comparing children’s reports with parental observations regarding gameplay frequency and tobacco-related interactions.

Interviews were conducted either face-to-face in private settings (*n* = 12) or via secure video conferencing platforms (*n* = 30), each lasting approximately 30–40 min. Participants received a small incentive for participation (around 20 RMB). All sessions were audio-recorded, transcribed verbatim in Chinese, and checked for accuracy by a second researcher. Field notes documented contextual factors and non-verbal cues and were integrated into the transcripts for analysis.

### Data Analysis

Thematic analysis was employed following Braun and Clarke’s six-phase process (Braun & Clarke, 2006). Coding used a combined deductive and inductive approach, with initial codes informed by the research questions and interview guide domains, and additional codes generated from repeated reading of the transcripts. Two researchers independently coded the transcripts through line-by-line analysis, compared and refined codes through discussion, and grouped recurring patterns into broader themes. To enhance validity, triangulation was used across three dimensions: (1) data-source triangulation compared conflicting accounts between children and parents regarding gameplay aspects; (2) investigator triangulation involved independent coding and consensus discussions to resolve discrepancies; and (3) methodological triangulation integrated interview data with demographic information from questionnaires. All analysis was performed in Chinese, with key quotations anonymized and translated into English, then back-translated by two bilingual researchers to ensure accuracy. Participant quotes are labeled as ‘C’ for children (e.g., C10, M=Child 10, Male) and ‘P’ for parents (e.g., P10, F=Parent 10, Female). Data management and analysis were conducted using NVivo 12 software.

## Results

### Participant Characteristics

Table 1 summarizes the demographic characteristics of the 21 child-parent pairs. Children had a mean age of 10.3 years (SD = 1.7), with 81.0% in primary grades 4 to 6. Over half (57.1%) had played cigarette card games for 7 to 12 months. Parents were mainly mothers (81.0%), with a mean age of 39.1 years (SD = 6.3). Regarding educational background, 52.4% of parents completed secondary education, and 42.8% had attained tertiary education. Most families lived in urban areas (61.9%), followed by towns (23.8%) and rural areas (14.3%). Notably, 90.5% of households included at least one family member who smokes. Detailed characteristics for each child-parent pair are provided in Supplementary Table S1.

**Table 1:** Characteristics of participant and household (*N* = 21)

Children characteristics	*n* (%)
Sex	
Male	19 (90.5)
Female	2 (9.5)
Age, mean (SD)	10.3 (1.7)
Grades	
Primary 1–3	4 (19.0)
Primary 4–6	17 (81.0)
Cigarette card game history	
1–3 months	4 (19.0)
4–6 months	3 (14.3)
7–12 months	12 (57.1)
13–24 months	2 (9.5)
Parents characteristics	
Relation to child	
Father	4 (19.0)
Mother	17 (81.0)
Age, mean (SD)	39.1 (6.3)
Education attainment	
Primary	1 (4.8)
Secondary	11 (52.4)
Tertiary or above	9 (42.8)
Household characteristics	
Residence	
Urban	13 (61.9)
Town	5 (23.8)
Rural	3 (14.3)
Smoking family	
Non-smoking	2 (9.5)
Smoking	19 (90.5)

Note: SD - Standard Deviation

Based on the interviews, thematic analysis revealed four main themes.

### Chance and Reward in Gambling-like Engagement

Unlike conventional card games in which players simply compare hands or scores, cigarette card games require children to wager cards they already own, so that outcomes involve both the possibility of winning additional cards and the risk of losing valued ones. The reward therefore extends beyond winning a round itself to the acquisition of rare or high-value cards, which can also confer status among peers. Children indicated that their continued engagement with the game was driven not only by the collection of cards but also by the excitement of winning and the risk of losing, both of which contributed to a sense of intensity and gratification.

One participant described the compelling nature of the game, where the drive to win and outperform others provided a significant sense of accomplishment.

*“I love playing cigarette cards with my friends because winning gives me such a great sense of achievement that I just can’t get enough. I’m driven by the desire to beat others and score their rare cards.”* (C15, M).

Another child drew a direct comparison between cigarette cards and gambling chips, emphasizing how the value of each card symbolized both a win and a loss, akin to betting in a casino.

*“For me*,* cigarette cards are like chips in a casino. They represent winning or losing. When I win*,* it means you owe me back*,* and each card has a value*,* determined by its design rather than the card itself.”* (C12, M).

Parents also expressed concerns about the competitive nature of the game, particularly when it escalates beyond casual play. One parent articulated discomfort with the idea of her child actively competing for cards, drawing a parallel with gambling.

*“I don’t allow my child to truly compete for winning cards. It’s fine if they play casually*,* but when it becomes about really winning*,* it starts to feel like gambling*,* and that worries me.”* (P2, F).

### Peer Influence and Social Reinforcement

Children described cigarette card games as a shared activity shaped by peer interactions and group approval. Seeing classmates play often triggered curiosity and a compulsion to join in, with many noting that the game fostered a sense of inclusion and belonging. Beyond competing and winning cards, the gameplay served to cultivate friendships and strengthen social connections.

One child reflected on the peer pressure to participate, explaining that seeing classmates play creates an immediate sense of connection and a desire to belong.

*“When I see kids playing cigarette cards*,* it feels like they’re all already friends*,* and I want to join in too because everyone’s doing it. If I don’t*,* I feel like I might be left out.” *(C5, F).

Another child shared how playing cigarette card games with friends enhances social bonds and even helps forge new friendships.

*“Playing cigarette cards with my best friends not only brings us closer together but also helps me make new friends. It really improves my relationships with my classmates.”* (C11, M).

Parents echoed these observations, acknowledging that prohibiting participation seemed unrealistic given the game’s ubiquity among peers, which could lead to social exclusion or isolation for their child.

*“Anyway*,* I think they all play it*,* and when they gathered together*,* they often talk about cigarettes and the games. Anyway*,* we think not letting him have any engagement at all is unrealistic.” *(P2, F).

### Parental Misperceptions as a Harm-reduction Alternative

Many parents view cigarette card games as a safer alternative to screen-based entertainment. They often viewed the game as a safer alternative because it drew children outdoors, brought them together with peers, and involved more movement than screen-based activities. However, this perception often overlooked the potential for these games in promoting addictive behaviors, inadvertently normalizing elements of gambling.

One parent expressed a common viewpoint by emphasizing the relative safety of cigarette card games compared to electronic devices, viewing them as a more socially engaging and physically active option for children.

*“Electronic devices are far more harmful. When kids play cigarette cards*,* they’re interacting with classmates*,* and I don’t think it has much of a negative impact. It’s just like the games we played when we were young—it’s a mutual activity and doesn’t damage their eyes or anything.” *(P13, F).

Another parent recognized the potential benefits of cigarette card games in reducing screen time, seeing them as an opportunity for children to spend more time outdoors. She viewed the game as a harmless form of entertainment, provided it did not interfere with academic responsibilities.

*“I believe it’s much better for children to play cigarette card games than to be on their phones all the time. When they’re playing*,* they use their phones less and go out more often. As long as it doesn’t interfere with their studies*,* a little cigarette card play for fun is perfectly fine.”* (P1, F).

One parent initially viewed the game as beneficial to her child’s health because it appeared to encourage more outdoor activity and movement. However, she later became concerned as her child’s involvement grew excessive, suggesting that the game could be addictive.

*“He used to be quite chubby*,* but once he started playing and got involved in activities like kicking a ball and collecting cards*,* he lost weight and grew taller. I wouldn’t have restricted him if he hadn’t become too addicted later on.”* (P9, F).

### Parental Facilitation of Game Participation

Parental involvement in cigarette card games extended beyond passive approval to active facilitation. Some parents actively facilitated their children’s participation by ensuring access to cards and related items, often through the efforts of extended family, friends, and colleagues who helped to source the cards.

One parent recalled how she facilitated her child’s collection by reaching out to multiple acquaintances to obtain premium cigarette cards.

*“I contacted one friend and asked him to gather some cigarette cards for me*,* and even reached out to four or five others*,* so that his collection mostly consisted of top brands like Chunghwa (中华) and Furongwang (芙蓉王)*,*”she explained*. (P7, F)

Other parents described how they mobilized their extended family to ensure a steady supply of cards for their children, sometimes even receiving shipments from relatives in other cities.

*“He never collected cigarette cards himself. Some were even mailed by his grandmother from Shantou*,* while his dad*,* aunts*,* and grandparents all collected them at home so he always had enough.” *(P12, M).

One parent pointed out that without parental involvement, children would find alternative ways to acquire the cards. This involvement ensured that children were able to participate without feeling excluded.

*“When colleagues had cigarette boxes*,* I would take some for my child. If parents didn’t give them*,* children would find other ways to get these things*,* so it was better for us to bring them home directly. This way*,* he could join in at school instead of feeling left out.” *(P14, F).

## Discussion

This qualitative study provides new insights into how Chinese children and their families engage with cigarette card games, highlighting their addictive potential and the critical influence of parental attitudes and behaviors. Our findings reveal a multifaceted interplay of individual motivation, peer reinforcement, and familial facilitation, extending prior research on gambling-like behaviors and the normalization of risky play in childhood.

Gambling-related features embedded in cigarette card games evoke excitement and a sense of anticipation. The unpredictable outcomes and the perceived value of rare cards create a rewarding cycle that sustains engagement. This experiential “thrill” reflects the same psychological processes identified in gambling research, where chance, intermittent rewards, and uncertain gains reinforce repetitive play. Children in our study frequently described intense achievement and excitement tied to unpredictable wins of rare cards, with some explicitly compared the experience to adult gambling. Neurobiological research indicates that children’s developing reward systems may be especially sensitive to the dopaminergic highs produced by uncertain rewards (Imataka et al., 2022; Wang et al., 2020). Consistent with this evidence, the compulsive patterns we observed raise concerns about early behavioral conditioning and the potential progression toward problematic gaming or gambling tendencies in later life.

Peer influence emerged as a powerful force in sustaining participation (Savolainen et al., 2020) and encouraging risk-taking (Chein et al., 2011). The games not only provided entertainment but also a form of social currency that defined belonging and status within peer groups. Children described a strong pressure to join in to build friendships and avoid exclusion, suggesting that the games reinforce both inclusion and peer hierarchy (Albert et al., 2013). This pattern aligns with research on loot boxes and other random-reward systems, where unpredictability and social comparison drive repeated engagement among youth (Bestman et al., 2017; Drummond et al., 2020; Xiao et al., 2024). Elements of competition and wagering, such as “betting,” attempts to “outwit” opponents, and the experience of loss, parallel trajectories of loss-chasing and escalating involvement reported in youth gambling research (Botella-Guijarro et al., 2020; Rizzo et al., 2023; Riley et al., 2021). Specifically, social reinforcement in the games may heighten vulnerability among boys, who comprised most of our sample and frequently described stronger competitive motives. This finding suggests the importance of distinguishing peer influences in physical play from those in digital environments, as cigarette cards blend face-to-face interaction with exposure to tobacco imagery, potentially amplifying long-term behavioral and health risks.

While much attention has focused on children’s engagement, this study also highlights the pivotal yet often contradictory role of parents. Many parents viewed the games as a harmless pastime and even a healthier alternative to screen-based entertainment that encouraged physical activity and social interaction. Although well intentioned, this harm-reduction perspective may overlook how cigarette card games combine wagering, the loss of owned cards, and the pursuit of rare or valued cards, all of which can reinforce reward-seeking and loss-chasing (Viola et al., 2025; Yau & Potenza, 2015). Children did not necessarily frame these cards in explicit relation to smoking. However, repeated contact with cigarette packaging and branding during play may make tobacco-related imagery more familiar and less salient, even without conscious identification with smoking. Similar attitudes have been reported in studies of parental views on video gaming and gambling-themed features, where perceived social benefits mask potential harms (Canale et al., 2016; Krossbakken et al., n.d.; Viola et al., 2025). In Macau, for example, 81% of parents taught children gambling games despite disapproval (So et al., 2017), highlighting how facilitation often stems from perceived harmlessness. Culturally, Chinese parents distinguish between “real smoking” (forbidden) and “play” (tolerated). Viewing cigarette cards as traditional play items, they assume children focus on the game, not the branding. This explains why one parent called complete non-engagement “unrealistic.” Our study further reveals that some parents and extended family members actively facilitated participation by sourcing or supplying cigarette cards and mobilizing social networks to secure their children’s social inclusion at school. Such facilitation may create a bidirectional pathway of harm, as children’s demand for cigarette cards may also encourage adults to maintain or expand access to cigarette packs and related materials. Although direct evidence linking cigarette card play to later smoking remains limited, repeated engagement with cigarette-branded packaging during play may nonetheless normalize tobacco-related imagery and attitudes. The tobacco-related imagery involved in this process is illustrated in Fig. 1, where the cards prominently display brand logos, colors, and packaging elements. This possibility, together with the potential progression to other forms of gambling-like behavior, warrants further longitudinal research. This parallels evidence from youth gambling research showing that parental modeling and provision of gambling opportunities predict adolescent initiation (Zhai et al., 2017).

These findings call for coordinated public health action to counter growing normalization of gambling-like play and tobacco imagery in childhood. Policy and education responses should move beyond viewing cigarette card games as harmless recreation and instead recognize their potential to condition addictive reward responses and reinforce tobacco-related cues. Parent-focused educational programs should challenge misperceptions by highlighting the games’ gambling-related risks and the influence of tobacco branding on children’s attitudes and behavior. School-based programs can strengthen children’s resistance to peer pressure and promote critical reflection on reward-based play, while supporting families in developing healthier, balanced leisure routines. Future longitudinal and mixed-methods research are needed to track behavioral trajectories of gambling and smoking, identify vulnerable subgroups, and guide evidence-based prevention and policy interventions.

## Limitations

This study has several limitations. First, most participants were from urban families, so the findings may not capture the experiences of children in rural or different cultural settings. Second, the predominance of boys in the sample may limit the transferability of findings to girls, who might experience distinct peer relationships or parental expectations around play. Third, the overrepresentation of smoking households (90.5%) reflects China’s high smoking prevalence (approximately 50% of men smoke). Moreover, the snowball sampling bias, where participants tend to refer peers with similar characteristics, may also limit the transferability of our findings to non-smoking families. Finally, as a qualitative study, the findings are exploratory and hypothesis-generating rather than generalizable.

## Conclusion

Cigarette card games intertwined addictive gambling elements with tobacco normalization, amplified by parental pro-game practices. This underscores the urgent need for family education on gambling risks. Interventions should promote responsible play, balancing social benefits with harm reduction.

## Electronic Supplementary Material

Below is the link to the electronic supplementary material.

Supplementary Material 1

## Data Availability

Due to confidentiality agreements and to protect participant privacy, the qualitative data cannot be made publicly available. Data may be available from the corresponding author upon reasonable request.

## References

[CR1] Albert, D., Chein, J., & Steinberg, L. (2013). Peer Influences on Adolescent Decision Making. *Current Directions in Psychological Science*, *22*(2), 114–120. 10.1177/0963721412471347 25544805 PMC4276317

[CR2] Bestman, A., Thomas, S., Randle, M., & Pitt, H. (2017). Children’s attitudes towards Electronic Gambling Machines: An exploratory qualitative study of children who attend community clubs. *Harm Reduction Journal*, *14*(1), 20. 10.1186/s12954-017-0148-z 28482912 PMC5422940

[CR3] Botella-Guijarro, Á., Lloret-Irles, D., Segura-Heras, J. V., Cabrera-Perona, V., & Moriano, J. A. (2020). A Longitudinal Analysis of Gambling Predictors among Adolescents. *International Journal of Environmental Research and Public Health*, *17*(24), 9266. 10.3390/ijerph17249266 33322378 PMC7763018

[CR4] Braun, V., & Clarke, V. (2006). Using thematic analysis in psychology. *Qualitative Research in Psychology*, *3*(2), 77–101. 10.1191/1478088706qp063oa

[CR5] Canale, N., Vieno, A., Ter Bogt, T., Pastore, M., Siciliano, V., & Molinaro, S. (2016). Adolescent Gambling-Oriented Attitudes Mediate the Relationship Between Perceived Parental Knowledge and Adolescent Gambling: Implications for Prevention. *Prevention Science*, *17*(8), 970–980. 10.1007/s11121-016-0683-y 27448214

[CR6] Chan, K. H., Xiao, D., Zhou, M., Peto, R., & Chen, Z. (2023). Tobacco control in China. *The Lancet Public Health*, *8*(12), e1006–e1015. 10.1016/S2468-2667(23)00242-6 38000880

[CR7] Chein, J., Albert, D., O’Brien, L., Uckert, K., & Steinberg, L. (2011). Peers increase adolescent risk taking by enhancing activity in the brain’s reward circuitry. *Developmental Science*, *14*(2), F1–F10. 10.1111/j.1467-7687.2010.01035.x 21499511 PMC3075496

[CR8] Chen, T. (2024). November 28). China: Cigarette Card Game among Chinese Youth: A New Challenge for Tobacco Control. *Blog - Tobacco Control*. https://blogs.bmj.com/tc/2024/11/28/china-cigarette-card-game-among-chinese-youth-a-new-challenge-for-tobacco-control/

[CR9] Chen-Sankey, J. C., van de Venne, J., Westneat, S., Rahman, B., Folger, S., Anesetti-Rothermel, A., Debnam, C., Ribisl, K. M., Cohn, A., & Rose, S. W. (2022). Real-Time Context of Tobacco Marketing Exposure and Community Vulnerability-An Ecological Momentary Assessment Among Young Adults. *Annals of Behavioral Medicine*, *56*(6), 620–631. 10.1093/abm/kaab066 34323267 PMC9242544

[CR10] Drummond, A., Sauer, J. D., Hall, L. C., Zendle, D., & Loudon, M. R. (2020). Why loot boxes could be regulated as gambling. *Nature Human Behaviour*, *4*(10), 986–988. 10.1038/s41562-020-0900-3 32601458

[CR11] Ferrari, M. A., Chan, M., Brown, P. N., & Clark, L. (2018). Slot machine gambling and testosterone: Evidence for a winner-loser effect? *Psychology of Addictive Behaviors: Journal of the Society of Psychologists in Addictive Behaviors*, *32*(8), 961–971. 10.1037/adb0000425 30475015

[CR12] Gilman, S. E., Rende, R., Boergers, J., Abrams, D. B., Buka, S. L., Clark, M. A., Colby, S. M., Hitsman, B., Kazura, A. N., Lipsitt, L. P., Lloyd-Richardson, E. E., Rogers, M. L., Stanton, C. A., Stroud, L. R., & Niaura, R. S. (2009). Parental smoking and adolescent smoking initiation: An intergenerational perspective on tobacco control. *Pediatrics*, *123*(2), e274–281. 10.1542/peds.2008-2251 19171580 PMC2632764

[CR13] Imataka, G., Sakuta, R., Maehashi, A., & Yoshihara, S. (2022). Current Status of Internet Gaming Disorder (IGD) in Japan: New Lifestyle-Related Disease in Children and Adolescents. *Journal of Clinical Medicine*, *11*(15), 4566. 10.3390/jcm11154566 35956181 PMC9369635

[CR14] Jiang, J. (2024). Game of Cigarette Cards: Unveiling the Challenges to Youth Tobacco Exposure and Tobacco Control in China. *Nicotine & Tobacco Research: Official Journal of the Society for Research on Nicotine and Tobacco*, *27*(1), 159–160. 10.1093/ntr/ntae169 38995043

[CR15] Krossbakken, E., Torsheim, T., Mentzoni, R. A., King, D. L., Bjorvatn, B., Lorvik, I. M., & Pallesen, S. (n.d.). The effectiveness of a parental guide for prevention of problematic video gaming in children: A public health randomized controlled intervention study. *Journal of Behavioral Addictions*, *7*(1), 52–61. 10.1556/2006.6.2017.087 PMC6035025 29313731

[CR16] Lapierre, M. A., Fleming-Milici, F., Rozendaal, E., McAlister, A. R., & Castonguay, J. (2017). The Effect of Advertising on Children and Adolescents. *Pediatrics*, *140*(Suppl 2), S152–S156. 10.1542/peds.2016-1758V 29093052

[CR17] Lin, B., Xie, X., Chen, T., Lu, W., Li, M., Nan, Y., Xie, H., Cheung, Y. T. D., Wang, M. P., Luk, T. T., Xiao, L., & Xiong, J. (2025). Awareness and attitudes towards children’s cigarette card gaming behaviour among adults aged 15 and above in Shenzhen, China: A population representative cross-sectional study. *Tobacco Control*. 10.1136/tc-2024-059216. tc-2024-059216. 40436439

[CR18] Montini, T., George, A., Martin-Mollard, M., & Bero, L. A. (2010). The role of public participation in public health initiatives: An analysis of the WHO Framework Convention on Tobacco Control. *Global Public Health*, *5*(1), 48–61. 10.1080/17441690802164391 19326277

[CR19] Peters, E. N., Nordeck, C., Zanetti, G., O’Grady, K. E., Serpelloni, G., Rimondo, C., Blanco, C., Welsh, C., & Schwartz, R. P. (2015). Relationship of gambling with tobacco, alcohol, and illicit drug use among adolescents in the USA: Review of the literature 2000–2014. *The American Journal on Addictions*, *24*(3), 206–216. 10.1111/ajad.12214 25864783

[CR20] Pitt, H., Thomas, S. L., Bestman, A., Daube, M., & Derevensky, J. (2017). Factors that influence children’s gambling attitudes and consumption intentions: Lessons for gambling harm prevention research, policies and advocacy strategies. *Harm Reduction Journal*, *14*(1), 11. 10.1186/s12954-017-0136-3 28212685 PMC5316223

[CR21] Pollay, R. W. (2000). Targeting youth and concerned smokers: Evidence from Canadian tobacco industry documents. *Tobacco Control*, *9*(2), 136–147. 10.1136/tc.9.2.136 10841849 PMC1748318

[CR22] Reynolds, C. M. E., Hanafin, J., Sunday, S., McAvoy, H., & Clancy, L. (2023). Odds of gambling among adolescents: A secondary analysis of the cross-sectional European School Survey Project on Alcohol and Other Drugs. *Lancet (London England)*, *402*(Suppl 1). 10.1016/S0140-6736(23)02118-9 37997124

[CR23] Riley, B. J., Oster, C., Rahamathulla, M., & Lawn, S. (2021). Attitudes, Risk Factors, and Behaviours of Gambling among Adolescents and Young People: A Literature Review and Gap Analysis. *International Journal of Environmental Research and Public Health*, *18*(3), 984. 10.3390/ijerph18030984 33499418 PMC7908209

[CR24] Rizzo, A., La Rosa, V. L., Commodari, E., Alparone, D., Crescenzo, P., Yıldırım, M., & Chirico, F. (2023). Wanna Bet? Investigating the Factors Related to Adolescent and Young Adult Gambling. *European Journal of Investigation in Health Psychology and Education*, *13*(10), 2202–2213. 10.3390/ejihpe13100155 37887156 PMC10606462

[CR25] Savolainen, I., Kaakinen, M., Sirola, A., Koivula, A., Hagfors, H., Zych, I., Paek, H. J., & Oksanen, A. (2020). Online Relationships and Social Media Interaction in Youth Problem Gambling: A Four-Country Study. *International Journal of Environmental Research and Public Health*, *17*(21), 8133. 10.3390/ijerph17218133 33153222 PMC7663674

[CR26] So, E. M. T., Lao, Y. M. P., & Wong, I. L. K. (2017). Macau parents’ perceptions of underage children’s gambling involvement. *Asian Journal of Gambling Issues and Public Health*, *7*(1), 1. 10.1186/s40405-017-0021-8 28316902 PMC5334386

[CR27] Spicer, S. G., Fullwood, C., Close, J., Nicklin, L. L., Lloyd, J., & Lloyd, H. (2022). Loot boxes and problem gambling: Investigating the gateway hypothesis. *Addictive Behaviors*, *131*, 107327. 10.1016/j.addbeh.2022.107327 35397261

[CR28] Tone, S. (2024, December 6). *Starter Pack: How China’s Children Got Hooked on Cigarette Cards*. #SixthTone. https://www.sixthtone.com/news/1016302

[CR29] Tran, L. T., Wardle, H., Colledge-Frisby, S., Taylor, S., Lynch, M., Rehm, J., Volberg, R., Marionneau, V., Saxena, S., Bunn, C., Farrell, M., & Degenhardt, L. (2024). The prevalence of gambling and problematic gambling: A systematic review and meta-analysis. *The Lancet Public Health*, *9*(8), e594–e613. 10.1016/S2468-2667(24)00126-9 39025095

[CR30] Viola, E., Mehanović, E., Martorana, M., Renna, M., Sciutto, A., Giraudi, G., Ginechesi, M., Vullo, C., Vadrucci, S., Andrà, C., Ceccano, A., Casella, P., Faggiano, F., & Vigna-Taglianti, F. (2025). Parental correlates of adolescent gambling behavior: A study on 12–14 years old students in Italy. *Frontiers in Psychology*, *16*, 1563936. 10.3389/fpsyg.2025.1563936 41000510 PMC12458132

[CR31] Wang, M., Dong, H., Zheng, H., Du, X., & Dong, G. H. (2020). Inhibitory neuromodulation of the putamen to the prefrontal cortex in Internet gaming disorder: How addiction impairs executive control. *Journal of Behavioral Addictions*, *9*(2), 312–324. 10.1556/2006.2020.00029 32663381 PMC8939425

[CR32] Xiao, L. Y., Fraser, T. C., Nielsen, R. K. L., & Newall, P. W. S. (2024). Loot boxes, gambling-related risk factors, and mental health in Mainland China: A large-scale survey. *Addictive Behaviors*, *148*, 107860. 10.1016/j.addbeh.2023.107860 37729858

[CR33] Yau, Y. H. C., & Potenza, M. N. (2015). Gambling disorder and other behavioral addictions: Recognition and treatment. *Harvard Review of Psychiatry*, *23*(2), 134–146. 10.1097/HRP.0000000000000051 25747926 PMC4458066

[CR34] Zhai, Z. W., Yip, S. W., Steinberg, M. A., Wampler, J., Hoff, R. A., Krishnan-Sarin, S., & Potenza, M. N. (2017). Relationships between perceived family gambling and peer gambling and adolescent problem gambling and binge-drinking. *Journal of Gambling Studies*, *33*(4), 1169–1185. 10.1007/s10899-017-9670-x 28101835 PMC5515696

[CR35] Zhao, S. Z., Yin, H., Tu, J., Weng, X., & Wang, M. P. (2025). From packs to games: A qualitative study on children’s experiences and perceptions of cigarette card games in China. *Tobacco Control*. 10.1136/tc-2025-059342. tc-2025-059342. 40685325

