# Supplementary material for: Exploring Gambling-like Behaviors in Children’s Cigarette Card Games and Parental Pro-game Perceptions in China: A Qualitative Study
Source: J Gambl Stud. 2026 May 29;42(3):979–92. doi: 10.1007/s10899-026-10514-2 (PMC13578003; doi:10.1007/s10899-026-10514-2)
Supplement: Supplementary file 1 [file 10899_2026_10514_MOESM1_ESM.docx]

**Table S1 Demographic characteristics** **of child-parent dyads (N=21)**

| Family ID | Child age (years) | Child sex | Grade | Cigarette card game history | Parent age (years) | Relation to child | Parent education | Province | Residence^1^ | Smoking household^2^ |
| --- | --- | --- | --- | --- | --- | --- | --- | --- | --- | --- |
| 1 | 9 | Male | Primary 3 | 3 months | 38 | Mother | Secondary | Hebei | Town | Smoking |
| 2 | 6 | Male | Primary 1 | 3 months | 33 | Mother | Secondary | Hebei | Town | Smoking |
| 3 | 9 | Female | Primary 4 | 12 months | 32 | Mother | Tertiary or above | Guangxi | Town | Smoking |
| 4 | 14 | Male | Primary 6 | 24 months | 49 | Father | Tertiary or above | Fujian | Urban | Smoking |
| 5 | 12 | Female | Primary 6 | 6 months | 42 | Mother | Tertiary or above | Fujian | Urban | Non-smoking |
| 6 | 10 | Male | Primary 5 | 9 months | 43 | Mother | Secondary | Sichuan | Urban | Smoking |
| 7 | 10 | Male | Primary 5 | 7 months | 37 | Mother | Tertiary or above | Shanxi | Urban | Smoking |
| 8 | 11 | Male | Primary 6 | 12 months | 33 | Mother | Tertiary or above | Sichuan | Rural | Smoking |
| 9 | 10 | Male | Primary 4 | 8 months | 34 | Mother | Tertiary or above | Hebei | Urban | Smoking |
| 10 | 11 | Male | Primary 5 | 8 months | 37 | Mother | Secondary | Hebei | Urban | Smoking |
| 11 | 12 | Male | Primary 6 | 24 months | 47 | Mother | Primary | Anhui | Urban | Smoking |
| 12 | 9 | Male | Primary 2 | 10 months | 45 | Father | Secondary | Guangdong | Rural | Smoking |
| 13 | 11 | Male | Primary 6 | 12 months | 40 | Mother | Secondary | Shanxi | Urban | Smoking |
| 14 | 9 | Male | Primary 4 | 6 months | 49 | Mother | Secondary | Henan | Urban | Non-smoking |
| 15 | 10 | Male | Primary 5 | 2 months | 39 | Mother | Secondary | Shanxi | Urban | Smoking |
| 16 | 12 | Male | Primary 6 | 12 months | 31 | Father | Tertiary or above | Sichuan | Urban | Smoking |
| 17 | 12 | Male | Primary 6 | 12 months | 45 | Father | Secondary | Shanxi | Urban | Smoking |
| 18 | 12 | Male | Primary 6 | 2 months | 38 | Mother | Tertiary or above | Shanxi | Town | Smoking |
| 19 | 9 | Male | Primary 2 | 7 months | 35 | Mother | Tertiary or above | Hebei | Urban | Smoking |
| 20 | 9 | Male | Primary 4 | 6 months | 28 | Mother | Secondary | Sichuan | Rural | Smoking |
| 21 | 10 | Male | Primary 5 | 12 months | 47 | Mother | Secondary | Shandong | Town | Smoking |

Note:

^1^ Based on participants’ self-reported residence: urban (cities), town (county-level towns), and rural (villages).

^2^ “Smoking” refers to families with at least one household member who smokes.
